# Hepatitis Virus in Long-Fingered Bats, Myanmar

**DOI:** 10.3201/eid1904.121655

**Published:** 2013-04

**Authors:** Biao He, Quanshui Fan, Fanli Yang, Tingsong Hu, Wei Qiu, Ye Feng, Zuosheng Li, Yingying Li, Fuqiang Zhang, Huancheng Guo, Xiaohuan Zou, Changchun Tu

**Affiliations:** Academy of Military Medical Sciences, Changchun, People’s Republic of China (B. He, F. Yang, Y. Feng, Y. Li, H. Guo, X. Zou, C. Tu);; Center for Disease Control and Prevention of Chengdu Military Region, Kunming, People’s Republic of China (Q. Fan, T. Hu, W. Qiu, Z. Li, F. Zhang)

**Keywords:** hepatitis, bats, bat hepatitis virus, full genome, viral morphology, prevalence, viruses, Myanmar

## Abstract

During an analysis of the virome of bats from Myanmar, a large number of reads were annotated to orthohepadnaviruses. We present the full genome sequence and a morphological analysis of an orthohepadnavirus circulating in bats. This virus is substantially different from currently known members of the genus *Orthohepadnavirus* and represents a new species.

The family *Hepadnaviridae* comprises 2 genera (*Orthohepadnavirus* and *Avihepadnavirus*), and viruses classified within these genera have a narrow host range. The genus *Orthohepadnavirus* consists of pathogens that infect mammals, and it currently contains 4 species: *hepatitis B virus*, *woodchuck hepatitis virus*, *ground squirrel hepatitis virus*, and *woolly monkey hepatitis B virus*. The genus *Avihepadnavirus* contains 2 avian species: *duck hepatitis B virus* and *heron hepatitis B virus* (*1*). Hepadnaviruses mainly infect the liver cells of their hosts and, in humans, cause hepatitis B, cirrhosis, and hepatocellular carcinoma (*2*). Approximately 2 billion persons worldwide are infected with hepatitis B virus (HBV), and 600,000 persons die every year from the consequences of hepatitis B (*3*).

Bats are associated with an increasing number of emerging and reemerging viruses, many of which pose major threats to public health (*4*). We conducted a viral metagenomic analysis of 6 species of bats from Myanmar. The analysis revealed a large number of viral contigs annotated to orthohepadnavirus with <70% nt identity (B. He, unpub. data), suggesting the presence of orthohepadnaviruses in these animals. We describe the virus by full genomic analysis and morphologic observation.

## The Study

We purchased 853 freshly killed insectivorous bats in Sedon and Wutao Counties in southeastern Kachin State, Myanmar; the counties are adjacent to Yunnan Province, People’s Republic of China. The bats covered 6 species: *Miniopterus fuliginosus* (n = 640), *Hipposideros armiger* (n = 8), *Rhinolophus ferrumequinum* (n = 176), *Myotis chinensis* (n = 11), *Megaderma lyra* (n = 6), and *Hipposideros fulvus* (n = 12). All bat tissue samples were subjected to viral metagenomic analysis (unpublished data). The sampling of bats for this study was approved by the Administrative Committee on Animal Welfare of the Institute of Military Veterinary, Academy of Military Medical Sciences, China.

We used PCR to further study the prevalence of orthohepadnavirus in the 6 bat species; the condition of the samples made serologic assay and pathology impracticable. Viral DNA was extracted from liver tissue of each of the 853 bats by using the QIAamp DNA Mini Kit (QIAGEN, Hilden, Germany). To detect virus in the samples, we conducted PCR by using the TaKaRa PCR Kit (TaKaRa, Dalian, China) with a pair of degenerate pan-orthohepadnavirus primers (sequences available upon request). The PCR reaction was as follows: 45 cycles of denaturation at 94°C for 30 s, annealing at 54°C for 30 s, extension at 72°C for 40 s, and a final extension at 72°C for 7 min. Positive results were obtained for 22 long-fingered bats (*Miniopterus fuliginosus*). Of these bats, 2.19% (7/320) were from Sedon County and 4.69% (15/320) from Wutao County; the viruses they harbored shared >98% nt identity. No other species had positive amplification results, indicating that *M. fuliginosus* was the most likely species to harbor orthohepadnaviruses.

**Figure 1:**
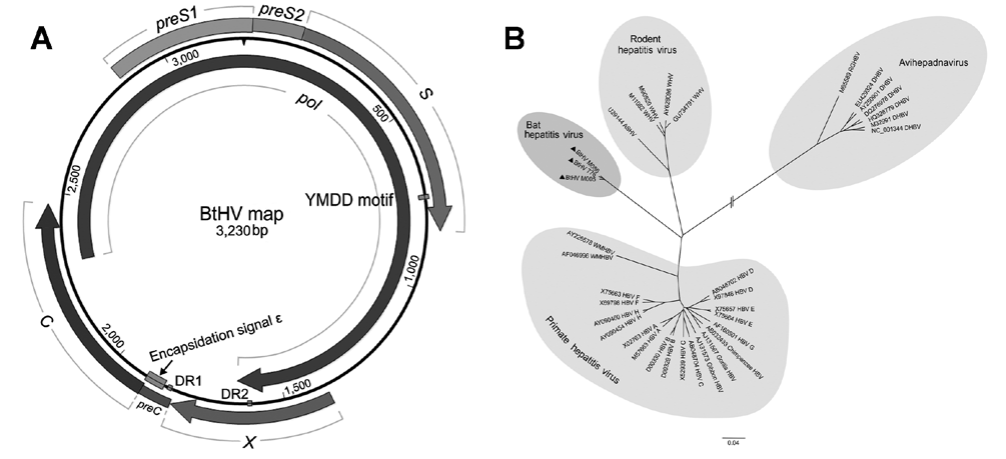
Predicted schematic representation of the bat hepatitis virus (BtHV) genome and its phylogenetic relationship with other hepadnaviruses. A) Genomic structural map of BtHV. Boxes and arrows represent the open reading frames encoding the main proteins: *pol* gene (2,305–1,636), *preS1/S2* and *S* gene (2,864–833), *preC/C* gene (1,815–2,468) and *X* gene (1,378–1,812). Two 12-nt direct repeat sequences (DR1 from 1,825 to 1,836 and DR2 from 1,594 to 1,605), the encapsidation signal ε (1,848–1,903), and YMDD domain (734–745) are also depicted in the map. B) Phylogenetic analysis of BtHVs and other hepadnaviruses based on amino acid sequences of *pol* genes. Representatives of hepadnavirus species belonging to *Orthohepadnavirus* and *Avihepadnavirus* genera were used; their GenBank accession nos. are shown in the trees. The different genotypes of human hepatitis B virus are also included. The 3 BtHV isolates are identified by black triangles. Scale bar indicates nucleotide substitutions per site.

Of the 22 positive samples, 3 were randomly selected for full genome amplification: M086 from Sedon County and 776 and M005 from Wutao County. PCR was conducted by using the PCR protocol defined above with high-fidelity *Pfu* DNA polymerase (Promega, Madison, WI, USA) and 4 pairs of specific primers (sequences available upon request). Four overlapping amplicons were obtained, sequenced in both directions, and assembled into the full genomic sequence by using SeqMan, version 7.1.0 (DNASTAR, Madison, WI, USA). All 3 full genomes (GenBank accession nos. JX941466– JX941468) were 3,230 nt in length, which is close to the size of primate hepatitis viruses (≈3,200 nt) but smaller than rodent hepatitis viruses (≈3,300 nt). We analyzed the genome structure by using Vector NTI Advance 10 (Invitrogen, Carlsbad, CA). The results showed that the bat hepatitis viruses (BtHVs) contained the same circular and compact genomic structure as other orthohepadnaviruses, comprising 4 open reading frames encoding the multifunctional Pol, preS1/preS2/S, preC/C, and X proteins in the same direction (Figure 1, panel A).

Genomic sequence comparison and phylogenetic analysis based on amino acids of the *pol* gene (2,562 bp) were constructed with ClustalW version 2.0 (www.clustal.org/) and MEGA5 (*5*). Phylogenetic tree analysis showed that previously described orthohepadnaviruses formed 2 clusters, primate hepatitis viruses and rodent hepatitis viruses, whereas the 3 newly identified BtHVs formed an independent cluster within the *Orthohepadnavirus* genus (Figure 1, panel B). Sequence comparison showed that the full genomes of the BtHVs were 63.1%–65.3% and 33.9%–34.8% identical to members of the *Orthohepadnavirus* and *Avihepadnavirus* genera, respectively. Similar low identities were also observed separately in the 4 genes of the BtHVs (Table). These results support the classification of the BtHVs within the *Orthohepadnavirus* genus, being distantly related to current species and likely to form a new species designated as BtHV.

**Table:** Gene lengths and percentage identity between bat orthohepadnavirus and other hepadnaviruses*

Virus†	*pol* gene					*preS1/preS2/S* gene					*preC/C* gene					*X* gene			
	nt	% ID	aa	% ID		nt	% ID	aa	% ID		nt	% ID	aa	% ID		nt	% ID	aa	% ID
BtHV776	2562	–	853	–		1200	–	399	–		654	–	217	–		435	–	144	–
HBV	2532	63	843	57		1203	63	400	59		639	65	212	66		465	61	154	49
WMHBV	2508	63	835	55		1176	64	391	60		636	65	211	63		459	66	152	50
WHV	2640	66	879	56		1281	66	426	51		678	69	225	71		426	67	141	44
ASHV	2634	67	877	53		1284	67	427	52		654	68	217	71		417	69	138	52
DHBV	2526	41	841	30		1104	43	367	30		888	42	295	22		NA	–	NA	–

*nt, nucleotide length; % ID, percentage identity of nt and amino acid sequence between BtHV and other viruses; aa, amino acid length; BtHV, bat hepatitis virus; –, not applicable; HBV, hepatitis B virus; WMHBV, woolly monkey HBV; WHV, woodchuck hepatitis virus; ASHV, arctic squirrel hepatitis virus; DHBV, duck HBV; NA, not available. †GenBank accession nos. for HBV, WMHBV, WHV, ASHV, and DHBV are D00329, AF046996, AY344076, U29144, and EU429324, respectively.

**Figure 2:**
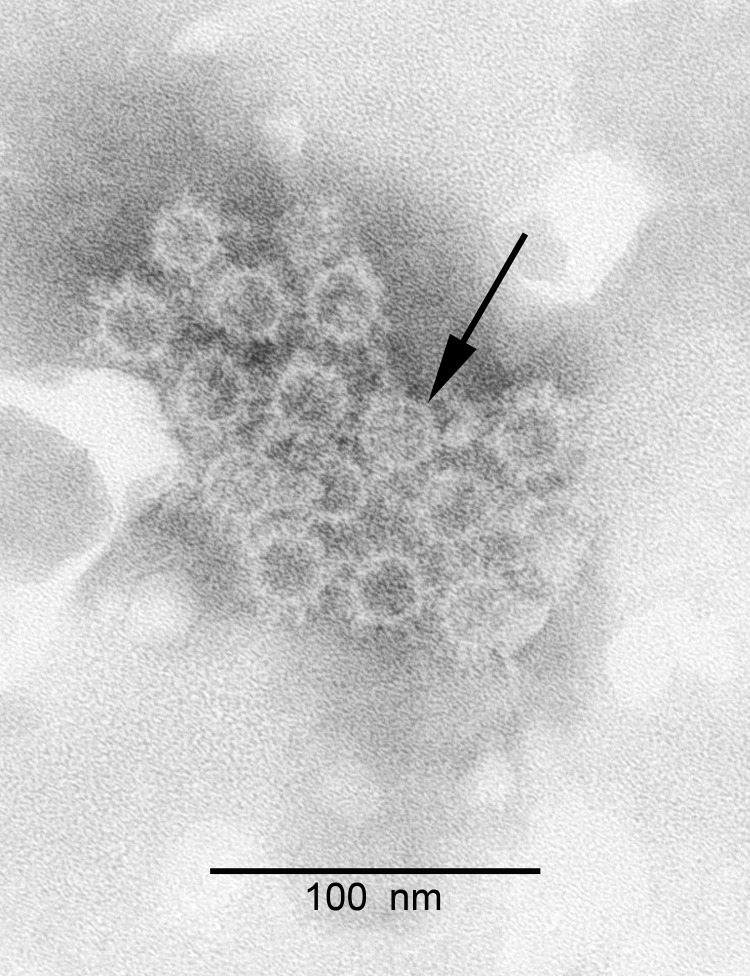
Electron microscopy of negative-stained orthohepadnavirus particles from a bat (arrow). Clumps of Australia antigen–like particles are seen.

Hepadnaviruses have not been grown in any available in vitro cell system; thus, we did not attempt to isolate BtHV in cell culture. To detect the presence of virus particles, we used pooled liver tissues from the 3 bats that were randomly selected for full genome amplification. We homogenized the pooled tissues in SM buffer (50 mM Tris, 10 mM MgSO_4_, 0.1M NaCl; pH7.5), followed by clarification by low-speed centrifugation to remove cell debris. We then passed the pooled sample through a 0.22-μm syringe filter (Millipore, Carrigtwohill, Ireland). Polyethylene glycol 6000 was added, and the resulting precipitate was sedimented at 12,000 × *g* in a desktop centrifuge (Eppendorf, Hamburg, Germany) for 40 min at 4°C. The pellet was resuspended and examined after negative staining in a JEM-1200 EXII transmission electron microscope (JEOL, Tokyo, Japan). Numerous spherical particles of ≈20 nm diameter were observed (Figure 2). The particles were morphologically similar to the Australia antigens of HBV, the most abundant viral component found in HBV-infected humans and animals and also known as surface protein or S antigen (*6*,*7*). PCR amplification of DNA extracted from the virus pellet revealed the full genome of the BtHV, with the expected size of ≈3200 bp (image not shown).

## Conclusions

Our observations provide strong evidence for the circulation of orthohepadnaviruses in at least 1 species of bats, *M. fuliginosus*, in Myanmar. These bats have a wide distribution (*8*), and increasing numbers of viruses, including coronaviruses and betaherpesviruses, are being isolated from them (*9*,*10*). Of the 6 bat species we sampled, only *M. fuliginosus* was positive for BtHV. The prevalence of BtHV-positive bats in the 2 counties from which we obtained bats, was 2.2% and 4.7%, respectively, indicating that this species is likely a natural reservoir host of BtHV. The lack of detection of BtHV in bats from the other 5 species may be due to the limited numbers of bats sampled (although no evidence of hepadnavirus was found in any of the 176 *R. ferrumequinum* bats) or to a narrow host range of the virus. Further study is required to determine the tropism and prevalence of BtHVs in other bat species.
